# Psychometric analysis and adaptation of the generalized anxiety questionnaire (GAD-7) to the Collao Quechua language in Peru

**DOI:** 10.17843/rpmesp.2024.412.13373

**Published:** 2024-06-13

**Authors:** Julio Cjuno, Raúl Alexis Villegas-Mejía, Jovita Coronado-Fernández

**Affiliations:** 1 Universidad Peruana Unión, Graduate School of Engineering and Architecture. Lima, Peru Universidad Peruana Unión Universidad Peruana Unión Graduate School of Engineering and Architecture Lima Peru; 2 Universidad Católica Los Ángeles de Chimbote, Nursing School. Chimbote, Peru Universidad Católica de Los Ángeles de Chimbote Universidad Católica Los Ángeles de Chimbote Nursing School Chimbote Peru

**Keywords:** Anxiety disorder, anxiety, GAD-7, indigenous peoples

## Abstract

**Objectives.:**

To adapt the 7-item Generalized Anxiety Questionnaire (GAD-7) from English to the cultural and linguistic context of Quechua Collao and to analyze its psychometric properties in Puno, Peru.

**Material and methods.:**

The GAD-7 in its original version was translated into the Collao Quechua variety and its psychometric properties were analyzed. The participants were bilingual (Spanish and Quechua), over 18 years of age and of both sexes. The exploratory factor analysis (EFA) was evaluated using parallel analysis, confirmatory factor analysis (CFA) and goodness-of-fit indices; reliability was also analyzed using McDonald’s classic alpha and Omega.

**Results.:**

Judges and focus group participants conducted the cultural and linguistic adaptation of the GAD-7 to Quechua Collao; the EFA reported the presence of a single factor (KMO=0.88, p=0.01); while the CFA confirmed adequate adjustments in the unifactorial model (CFI=0.994; TLI=0.991; SRMR=0.027; RMSEA=0.092), good reliability (α=0.896; ω=0.894) and was also invariant across age groups, sex, marital status and educational level.

**Conclusions.:**

The questionnaire showed validity for a unidimensional model of the GAD-7 adapted to the Collao Quechua, as well as optimal reliability and invariance by the evaluated groups. Its use could benefit anxiety research and care.

## INTRODUCTION

Anxiety is a common mental health disorder with a constant growth rate, and together with depression, affects more than 264 million people in the world ^1^. In the region of the Americas, 2.1% of the inhabitants suffer from anxiety; while prevalence rates of 5.3% have been reported in Peru ^2^^)^ and 16.2% in populations from the Peruvian highlands ^3^. In spite of being a frequent mental health problem, it is usually not diagnosed in time and treatment is almost always delayed, due to a lack of personnel with a command of the language and adequate instruments ^4^. An example of this is the Quechua, a native population in Peru, who despite having a large population of approximately 1,326,454 inhabitants ^5^, do not have anxiety screening instruments contextualized to their cultural and linguistic environment ^6^.

In this sense, it is important to highlight that the General Anxiety Disorder (GAD-7) has been widely used by different psychometric studies and is considered as a promising instrument for anxiety screening ^7^, because its theoretical construction was based on the diagnostic criteria of the DSM-IV for general anxiety ^8^. It was designed in English by a team of researchers from the United States ^9^. Currently, it has adaptations that make possible its use in the screening of anxiety, due to several adaptation and validation studies in languages such as English ^10^, German ^11^, Mandarin Chinese ^12^, Spanish ^13^, Portuguese ^14^, Bolivian Spanish ^15^, Colombian Spanish ^16^. In all previous studies, the GAD-7 has shown single-factor behavior and adequate evidence of validity and reliability. 

In Peru, the unifactorial model of the GAD-7 can be used in Spanish-speaking pregnant women ^17^^)^ and in young university students ^18^. The absence of anxiety measurements within Quechua-speaking communities in Peru creates a gap in the understanding of mental health in this population, which represents about 13.6% of the country’s total population ^19^. The lack of an adaptation to Quechua limits the ability to identify and address mental health problems within these communities and hinders the development of effective and culturally sensitive interventions for anxiety. Given the importance of mental health in people’s quality of life and well-being ^20^, it is essential to conduct studies that consider the experience and expression of anxiety in Quechua-speaking communities in order to ensure comprehensive and equitable care for all ethnic groups in the country.

Therefore, this study aimed to adapt the GAD-7 from English to the cultural and linguistic context of the Collao Quechua and to analyze its psychometric properties such as internal structure validity and reliability.

**KEY MESSAGES**

Motivation for the study. Peru has the largest Quechua population; however, the measurement of anxiety is not validated in the cultural and linguistic context of the Collao Quechua variety mainly spoken in Puno.

Main findings. The adaptation of the 7-item Generalized Anxiety Questionnaire (GAD-7) to Collao Quechua reported adequate internal validity, invariance, and optimal reliability.

Implications. The GAD-7 adapted to Collao Quechua could be implemented in primary health care for screening for generalized anxiety symptoms.

## MATERIAL AND METHODS

### Design and context

This was a psychometric study, developed in the Puno region of Peru, which is home to approximately 538,127 Quechua speakers of the Quechua Collao variety (57.0% of the population of Puno), who mainly engage in commercial activities, tourism, agriculture and livestock ^5^.

### Translation

The original English version of the GAD-7 was translated directly into the Collao Quechua variety. Once the translation was completed, the two translators and a Quechua-speaking researcher met to discuss the differences in the translations. Once the discrepancies were resolved and the translations unified, they proceeded with the back translation from Quechua to English. Subsequently, the translators and the research team verified the back translation along with the first translation, fine-tuning details and giving their approval to the final version of the Quechua Collao GAD-7.

### Cultural adaptation into Collao Quechua

By applying the Delphi method, we elaborated a cultural adaptation form (Supplementary Material: Annex 1) with some open questions for uncommon words in Quechua such as: “anxiety” and “nervous”. Additionally, we inquired about the change of the response category “more than half the days” to “ashka p’unchaykuna” and about the relationship between the Collao Quechua GAD-7 with the DSM-V for the diagnosis of generalized anxiety. Those questions were sent via e-mail. Three bilingual (i.e., Spanish and Quechua-speaking) psychologists participated, all of whom had at least one year of experience in the psychotherapeutic treatment of depression in Quechua-speaking patients of the Collao variety. The interaction between each expert and the research team took place in two rounds of emails, in addition to a meeting via Zoom with the research team in order to be able to assess suggestions and reach a consensus. 

Subsequently, we conducted a virtual focus group to verify the clarity and understanding of the items in the target population, this meeting lasted approximately 60 minutes. At the beginning, after obtaining informed consent, we asked the participants to answer the Collao Quechua GAD-7 in an online version shared via Google Forms. Then, the moderator (a native Quechua-speaking psychologist with knowledge of qualitative methods) invited the participants to give their opinion on the clarity and comprehension of the items in a language that was common and simple for Quechua speakers. Five Quechua speakers over the age of 18 (three females and two males) who were bilingual (Quechua and Spanish) participated, this process is shown in the (Supplementary Material: Appendix 2).

### Participants

The study had two samples. The final version of the GAD-7 translated into Collao Quechua was applied to a non-probabilistic sample of 206 participants for the exploratory factor analysis (EFA), following the recommendations for factor analysis of 20 participants per item, that is, 7 items X 20 participants each resulting in 140 participants ^21^. On the other hand, the sample included 454 participants for the confirmatory factor analysis (CFA), this number being higher than the number calculated by the application: https://wnarifin.shinyapps.io/ss_sem_cfi_equal/, where the sample size of the CFA for structural equation modeling (SEM) expected a sample of 414 participants, using a statistical power of 95% and a loss rate of 10%. 

Both samples included participants over 18 years of age, of both sexes, living in urban and rural settings, located in the departments of Puno and Cusco, who reported being bilingual Quechua speakers (Quechua and Spanish), with sufficient academic background to read Quechua (e.g., at least incomplete primary school), excluding speakers of other varieties of Quechua and those who did not provide informed consent.

### Instrument

The original English version of the GAD-7 ^22^, consists of 7 items that correspond to the generalized anxiety symptoms of the DSM-IV ^8^. Its response options evoke the frequency of occurrence of such symptoms in the last two weeks, considering the following Likert-type scale: 0 = not at all, 1 = several days, 2 = more than half the days, 3 = nearly every day. This defines a raw score between 0 and 21. The English version of the GAD-7 showed a sensitivity and specificity of 86% and 82% respectively, it also showed validity based on the relationship with other variables such as Beck’s Anxiety (r = 0.72) and the anxiety subscale of the Symptom Checklist-90 (r = 0.74) indicating external validity; likewise, it showed a good reliability of α = 0.92.

### Procedures

For data collection, two surveyors, who were fifth-year psychology students, were trained in the use and application of the instrument. The surveyors identified WhatsApp groups of parents from educational institutions, Christian churches and groups of peasant community associations to whom they presented the survey in Google Forms format. The informed consent form was included at the beginning of the survey and only those who agreed to participate in the study were able to respond to it. Data collection began in January and ended in February 2023.

### Statistical analysis

We conducted a descriptive analysis of the items (mean, standard deviation, skewness and kurtosis). Subsequently, we used the Exploratory Factor Analysis (EFA) to verify the number of factors through parallel analysis, additionally the Kaiser-Meyer-Olkin (KMO) test was estimated through the strength of the partial correlations between variables considering a KMO > 0.80 as adequate plausibility; additionally, Bartlett’s sphericity test (p < 0.05) should affirm that the correlation matrix is not an identity matrix ^23^. 

We also conducted a Confirmatory Factor Analysis (CFA) of the unidimensional model using a WLSMV (Weighted Least Square Mean and Variance Adjusted) estimator, as we did previously ^24^. We report the standardized betas of the model and standard goodness-of-fit measures: the X2 for model versus baseline, considering values < 3 acceptable; the comparative fit index (CFI), which is adequate when > 0.90; the Tucker-Lewis Index (TLI), which is acceptable when > 0.90. Likewise, we used the standardized root mean square residual (SRMR) and the root mean square error of approximation (RMSEA), considering them adequate with values ≤ 0.08 ^25^. SEM Structural Equation Modeling was used to graphically present the factor loadings ^26^^)^ and finally, internal consistency was estimated using the classic Alpha and McDonald’s Omega ^27^.

Measurement invariance was analyzed by means of the Multigroup CFA method considering groups defined by sex and marital status. The difference in CFI (ΔCFI) or RMSEA (ΔRMSEA) the criterion for determining models with more restrictions versus those with fewer restrictions, initially assuming the invariance configured as the base model, scaling to metric, strong and strict invariance. In order to determine an adequately constrained and appropriate model at each invariance stage the ΔCFI or ΔRMSEA had to have values < 0.01 ^28^.

Additionally, MIMIC (Multiple Indicators and Multiple Causes) models were used to determine invariance by age and educational level (e.g., because they were numerical variables or when it was not feasible to use the Multigroup CFA). Therefore, the invariance of the intercepts of the indicators and the mean differences of the latent dimensions were evaluated, all through groups according to the aforementioned variables. Each covariate was evaluated separately, each comparing two types of models; the first was a saturated version where the covariate explains all the observed items, but not the latent dimensions, and the second, a version of the intercept invariance model where the covariate explained all the latent dimensions, but not the items. To determine a more restricted model we had to identify a ΔCFI or a ΔRMSEA <0.01 ^28^. The statistical software R Studio version 3,3,0+ and its psych packages, GPArotation, polycor, lavaan, semTools, and semPlot were used for the analysis.

### Ethical considerations

This study was evaluated and approved by the Ethics Committee of the Universidad Peruana Unión with report number 2022-CE-FCS - UPeU-059. Likewise, all the ethical principles of research in human beings stated in the Declaration of Helsinki ^29^ were respected.

## RESULTS

### Cultural adaptation phase

The experts who participated in the cultural adaptation by the Delphi method provided valuable suggestions that contributed to improve the adaptation of the word "anxiety" which was initially adapted as “phutisk’alla”, then the experts suggested to add “ansiedad nisqhawan” which, although is a combination between Spanish and Quechua, helps to achieve better understanding; therefore, the final term was “phutisk’alla/anxiety nisqhawan” in item 1. On the other hand, the expression “nervous” was changed to “Ancha mancharisqha” according to the recommendation by the experts. The focus group participants gave a favorable opinion regarding the clarity and comprehension of the Collao Quechua GAD-7, stating that it was clear and in accordance with their cultural context without any additional changes.

The response options (Likert type) of the Collao Quechua GAD-7 were also changed by the expert judges and the focus group participants, because the category “more than half of the days” presented clarity problems in its translation. The team’s final recommendation was to use the expressions “Mana hayk’aqpas”, “Wakin p’unchawkunalla”, “Ashka p'unchawkuna”, “Yaqa llapa p’un chawkuna”, representing the Spanish equivalent of “never”, “some days”, “several days”, “almost every day”. After improving the items and response categories of the Collao Quechua GAD-7, the experts who participated in the cultural adaptation, in a last interaction with the research team, rated each item with the maximum score (3/3 in a range from 0 to 3) for each content validity indicator such as: relevance, representativeness, clarity and cultural equivalence.

### Characteristics of the participants

The EFA sample consisted of 206 participants with an average age of 31 years, of whom 57.3% (118) were female, 6.1% (132) were single/widowed/divorced and 59.7% (123) reported having a university education; on the other hand, the CFA sample consisted of 454 participants with an average age of 32 years, where 57.7% (262) were female, 57.0% (259) reported being single/widowed/divorced and 40.7% (185) indicated having university studies (Supplementary material: Appendix 3).

### Exploratory factor analysis

**Figure 1:**
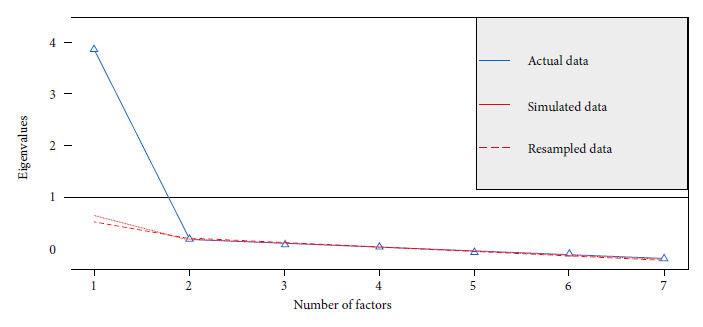
Parallel analysis of the GAD-7 adapted to Collao Quechua.

The sedimentation plot, or parallel analysis, reported the presence of a single latent factor (Figure 1). On the other hand, the Kaiser-Meyer-Olkin test and Bartlett’s test of sphericity (KMO = 0.88, p = 0.01) confirmed the presence of that factor from the included variables (Supplementary material: Appendix 3) with factor weights ranging from (0.64 - 0.85) obtained by the maximum likelihood method and varimax rotation; indicating that, theoretically, the items form a factor (Table 1).

**Table 1:** Descriptive analysis of the GAD-7 items adapted to Collao Quechua.

Items	Mean	Standard deviation	Asymmetry	Kurtosis	Factor loading (1 factor)
Question 1	0.78	0.81	0.87	0.30	0.65
Question 2	0.81	0.83	0.89	0.28	0.85
Question 3	0.93	0.80	0.76	0.40	0.75
Question 4	0.82	0.83	0.88	0.29	0.80
Question 5	0.69	0.83	1.16	0.85	0.66
Question 6	0.98	0.87	0.62	-0.28	0.64
Question 7	0.71	0.82	1.07	0.61	0.82

### Confirmatory factor analysis

**Figure 2:**
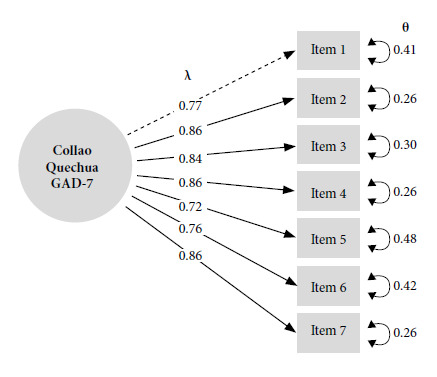
SEM graph of the GAD-7 unifactor model adapted to Col-laoQuechua with betas and errors.

The single factor model reported adequate goodness-of-fit values for the Collao Quechua GAD-7, (CFI = 0.994; TLI = 0.991; SRMR = 0.027; RMSEA = 0.092) (Table 2). Regarding factor loadings, a minimum of λ = 0.72 and a maximum of λ = 0.86 were obtained for the Collao Quechua GAD-7 items (Figure 2).

**Table 2:** Confirmatory Factor Analysis with WLSMV estimator for the GAD-7 adapted to Collao Quechua.

Model	Goodness of fit index	
1 dimension	X^2^ (14)	0.181
	Comparative Fit Index (CFI)	0.994
	Tucker-Lewis Index (TLI)	0.991
	Standardized Root Mean Square Residual (SRMR)	0.027
	Root Mean Square Error of Approximation (RMSEA)	0.092
	Alpha	0.896
	Omega	0.894

X^2^ (df>) Versus the baseline

### Reliability

Additionally, the Collao Quechua GAD-7 reported good reliability, with Cronbach’s Alpha values of 0.896 and Omega of 0.894 (Table 2).

### Measurement invariance

The multigroup CFA analysis for the Collao Quechua GAD-7 showed measurement invariance according to sex and marital status, presenting adequate goodness-of-fit indices (CFI > 0.98, RMSEA < 0.08) and values < 0.01 for at least the ΔCFI or ΔRMSEA; after verifying the configural invariance, we analyzed metric invariance (ΔCFI = 0.002), strong invariance (ΔCFI = 0.003) and strict invariance (ΔCFI < 0.004) (Table 3). On the other hand, by means of Multiple Indicator Multiple Causal Invariance (MIMIC), we confirmed the invariance by age and educational level since they presented adequate goodness-of-fit indices (CFI > 0.99, TLI > 0.99, RMSEA < 0.07, SRMR < 0.02) and all ΔCFI, ΔTLI, ΔMRSEA were < 0.01 in the saturated and intercept invariance (Table 4).

**Table 3:** Multigroup CFA invariance according to sex and marital status for the GAD-7 adapted to Collao Quechua.

Variable	Invariance	X^2^	df	p-value	CFI	Δ CFI	RMSEA	Δ RMSEA
Sex	1. Configural	31.742	28	-	0.992	-	0.079	-
	2. Metric	37.643	34	0.665	0.994	0.002	0.062	0.017
	3. Strong	52.986	47	0.028	0.991	0.003	0.064	0.002
	4. Strict	57.715	54	0.831	0.992	0.002	0.055	0.009
Civil status	1. Configural	30.754	28	-	0.992	-	0.078	-
	2. Metric	49.397	34	0.035	0.990	0.002	0.080	0.002
	3. Strong	47.144	47	1.000	0.993	0.003	0.057	0.023
	4. Strict	71.242	54	0.008	0.989	0.004	0.067	0.010

X² (gl) = Chi-square statistic (χ²) with degrees of freedom (df), CFI = Comparative adjustment index, Tli = Tucker-Lewis index, SRMR = Standardized residual of the average square root, RMSEA = Medium square error of Approximation, Δ = difference.

**Table 4:** Invariance of Multiple Indicators and Multiple Causes (MIMIC) by age and level of education

Covariates	Model	CFI	TLI	RMSEA	SRMR	Δ CFI	Δ TLI	Δ MRSEA
Age	Saturated MIMIC	0.994	0.991	0.068	0.027	-	-	-
	MIMIC intercept invariance	0.992	0.992	0.064	0.027	-0.002	0.001	-0.004
Education level	Saturated MIMIC	0.991	0.990	0.070	0.027	-	-	-
	MIMIC intercept invariance	0.994	0.990	0.069	0.027	0.003	0.000	-0.001

CFI = Comparative Fit Index, TLI = Tucker-Lewis Index, SRMR = Standardized Root Mean Square Residual, RMSEA = Root Mean Square Error of Approximation, Δ = difference.

## DISCUSSION

The GAD-7 adapted to Collao Quechua showed adequate adjustments for a unifactorial model, likewise it was invariant by sex, marital status, age and educational level; it also presented optimal reliability for its use in Quechua speakers of the Collao variety. The Collao Quechua GAD-7 could be useful for assessing anxiety symptoms in the Quechua-speaking population in the departments of Puno, Cusco, Arequipa, Moquegua, Tacna and Puerto Maldonado.

The graphical analysis of parallels and factor loadings in the EFA and the goodness-of-fit indices in the CFA supported a unifactorial model; the RMSEA presented somewhat elevated values but not to the point of reaching a mismatch ^30^. The RMSEA usually tolerates some mismatch due to the sample size ^31^. However, this behavior was not only reported by our study; studies conducted in Germany ^11^, Spain ^13^, Brazil ^14^, Colombia ^16^ and Bolivia ^15^ also found high values. Despite the high RMSEA trend in those studies, the RMSEA value reported by our study could be attributed to the size of the sample ^30^ and the differences in the proportions of subsamples, mainly by educational level. Another reason could be the inclusion of uneducated people, even though they could read, it is possible that some items are not easy for them to understand; further studies could seek to adapt the instrument in an audible format for native Quechua speakers without schooling. Despite this, the Collao Quechua GAD-7 is a valid screening instrument with a unifactorial structure because the other goodness-of-fit indices strictly met the expected parameters.

Our findings show optimal reliability of the scores derived from the Collao Quechua GAD-7 in the Peruvian population. This is consistent with other findings in previous GAD-7 validation studies such as Germany (α = 0.85) ^11^, China (α = 0.84) ^12^, Spain (α = 0.94) ^13^, Brazil (α = 0.92) (14), Colombia (α = 0.92) ^16^ who identified reliability values similar to those we found. In spite of all these studies being carried out in culturally and linguistically different populations, the measures of internal consistency by the classic alpha are similar. This allows for comparability of the intercultural anxiety variable by having achieved an optimal instrument in its internal consistency for Quechua-speaking populations of the Collao variety. The Classic Alpha was probably chosen to estimate reliability due to the search for comparability of the data for the purpose of meta-analysis ^32^. We additionally estimated the McDonald Omega with the aim of evaluating internal consistency from factor loadings, as it is more appropriate for estimating reliability ^27^, without being affected by the number of items or response categories, as is the case with the classical alpha ^33^. Having optimal reliability values shows that this version of the GAD-7 is consistent and reliable for measuring anxiety in Quechua speakers of the Collao variety, because the construct items are consistently correlated with each other in the measurement of the underlying construct, thus being able to offer reliable and consistent measurements.

The Collao Quechua GAD-7 also presented configural, metric, scalar and strict invariance for sex and marital status groups, and reported saturated and intercept invariance by age and educational level. Previous studies have reported similar results, such as the one conducted in Italy where the GAD-7 was invariant by age and gender ^34^; in China the GAD-7 showed evidence of invariance by age, gender, education level and residence ^35^; likewise, another study conducted in the United Kingdom, Ireland, Spain and Italy found saturated and intercept invariance by country, age and gender groups ^36^. Those similarities between previous studies and ours suggest that in Quechua speakers of the Collao variety, the GAD-7 Quechua Collao allows comparisons of the measure of anxiety without variation in the measurement between males and females, married/cohabiting and single/widowed/divorced, age group and in those with no education, with primary, secondary, higher technical and university education.

The present study has some limitations such as the self-report data collection technique, which could have influenced the participants to be dishonest when answering the questions; however, the participants were previously sensitized to the importance of being honest when answering the instrument. On the other hand, it is possible that the interviewer did not respect the self-reporting application protocol, and in some cases may have added explanations to the participants’ questions, which could have influenced the somewhat high RMSEA values; for this reason, we sought to control the effect of the evaluator with training in the presentation and data collection process, as well as constant monitoring of his/her role to ensure that the self-reporting collection technique was respected. It is also important to state that the MIMIC models we used for the assessment of invariance by age and educational level are limited to assessing the invariant intercept models and factor means, and assume that the rest of the structural and measurement parameters such as factor loadings, variance or covariance error, variance or covariance factor are the same across all levels of these variables.

In conclusion, our findings show validity for a unidimensional model of the Collao Quechua GAD-7, with optimal reliability and invariance by the evaluated groups such as age, sex, marital status and educational level. Its use could benefit research and care of patients with anxiety in the Quechua-speaking population from the Puno region of Peru. 
