# Supplementary material for: Psychometric analysis and adaptation of the generalized anxiety questionnaire (GAD-7) to the Collao Quechua language in Peru
Source: Rev Peru Med Exp Salud Publica. 2024 Jun 13;41(2):121–8. doi: 10.17843/rpmesp.2024.412.13373 (PMC11300701; doi:10.17843/rpmesp.2024.412.13373)
Supplement: Supplementary material. — Available in the electronic version of the RPMESP. [file rpmesp-41-02-13373-s001.pdf]

ANEXOS

ANEXO 1.

General Anxiety Disorder-7 (GAD-7) Quechua Collao

Kay ishkay qhepa semanakunapi, ¿Hayka kutitaq kay sasachaykunawan utaq llakikuykunawan karanki?

//Durante las últimas dos semanas, ¿con qué frecuencia ha sentido molestias por los siguientes problemas?//

**N°**	**Ítems**	**Mana hayk'aqpas** // Para nada//	**Wakin P'unchaykunalla** //Algunos días//	**Ashk'a p'unchaykuna** ///Varios días//	**Yak'a llapan p'unchaykuna** //Casi todos los días//
1	Ancha mancharisqha otaj phutisk'alla/ansiedad nisqhawan purishanki<br><br>//Sentirse nervioso, ansioso o con los pelos de punta//				
2	Manachu llakikuyta sak'eyta atinki/thaninki icha manachu controlayta atinki.<br><br>//No poder dejar de preocuparse o no poder controlar la preocupación//				
3	Nishutachu llakikunki imaymanakunamanta.<br><br>//Preocuparse demasiado por diferentes cosas//				
4	Sasachaywan samarikuyta tarirqhanki<br><br>//Dificultad para relajarse//				
5	Manatak tauq tiyarikuyta/ kausarikuyta atirankichu<br><br>//Estar tan inquieto que es difícil permanecer sentado o tranquilamente//				
6	Osqhayllachu phiñakunki otaj coleranky yanqhallamanta<br><br>//Molestarse o ponerse irritable fácilmente//				
7	Mancharikunkichu ima sasachakuypis/waqaypis hamushanman jina<br><br>//Sentir miedo como si algo terrible pudiera pasar//				

// ítems del GAD-7 versión español peruano//

ANEXO 2.

Figura 1.

Diagrama de flujo del proceso de traducción y adaptación cultural del inglés a quechua del GAD-7

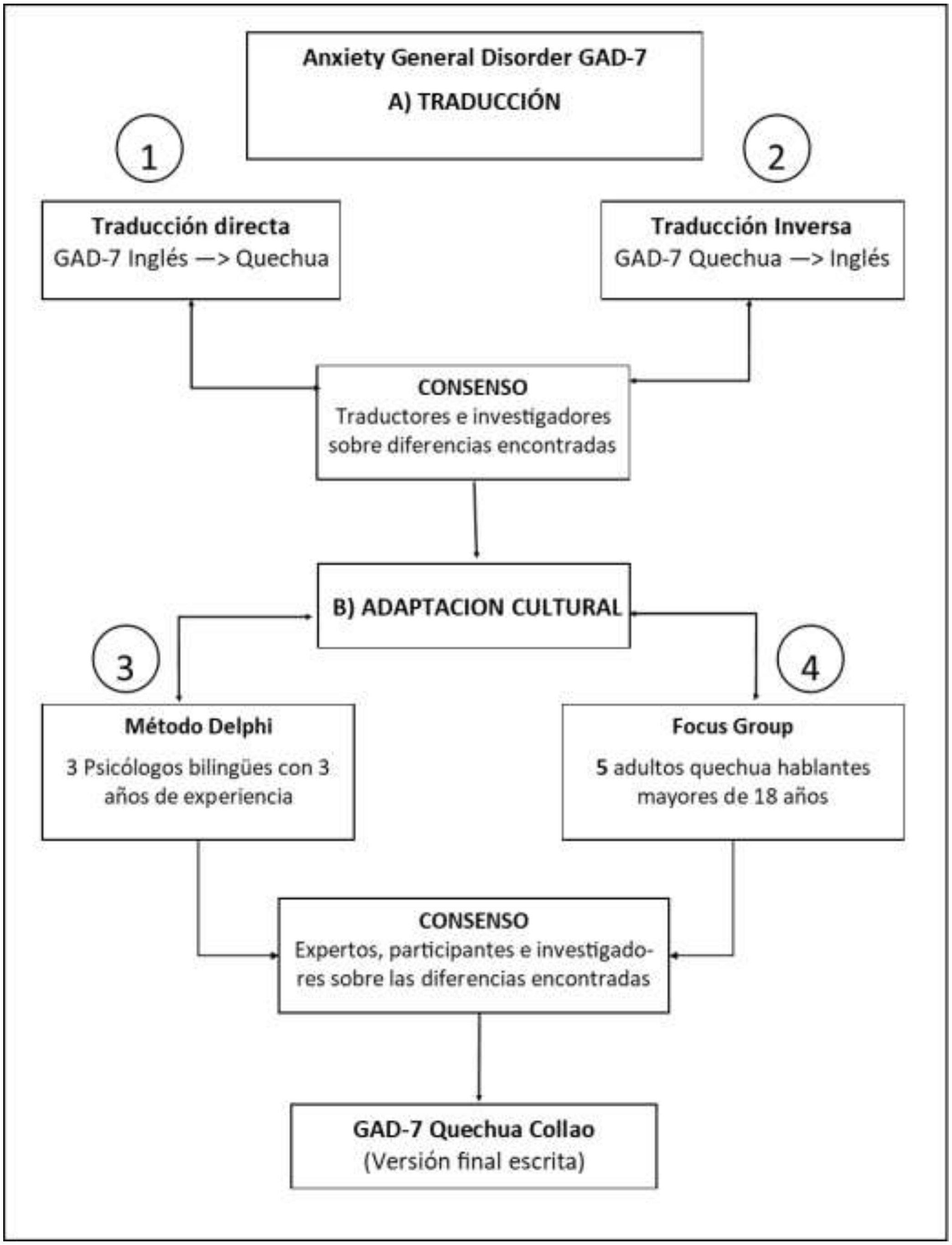

ANEXO 3.

Tabla 1.

Características de los quechua hablantes de la variedad Collao, Puno.

	**Muestra AFE (n=206)**		**Muestra AFC (n=454)**	
	Media	DE	Media	DE
**Edad**	31,2	12,3	32,7	14,0
	n	%	n	%
**Sexo**				
Femenino	118	57,3	262	57,7
Masculino	88	42,7	192	42,3
**Estado civil**				
Casado/Conviviente	74	35,9	195	43,0
Soltero/viudo/Divorciado	132	64,1	259	57,0
**Grado de instrucción**				
Sin estudios	3	1,5	28	6,2
Primaria	9	4,4	47	10,4
Secundaria	30	14,6	110	24,2
Superior técnico	41	19,9	84	18,5
Superior universitaria	123	59,7	185	40,7

DE, Desviación estándar, Muestra AFE= Muestra para el análisis factorial exploratorio, Muestra AFC= Muestra para el análisis factorial confirmatorio

Tabla 2.

KMO de los ítems del GAD-7 Quechua Collao

**ITEMS**	KMO
ítem 1	0,92
ítem 2	0,88
ítem 3	0,86
ítem 4	0,91
ítem 5	0,91
ítem 6	0,85
ítem 7	0,83
**KMO (total)**	0,88
**Bartlett-test (sig.)**	0,000

KMO, Kaiser-Meyer-Olkin
