# Supplementary material for: Psychometric analysis and adaptation of the generalized anxiety questionnaire (GAD-7) to the Collao Quechua language in Peru
Source: Rev Peru Med Exp Salud Publica. 2024 Jun 13;41(2):121–8. doi: 10.17843/rpmesp.2024.412.13373 (PMC11300701; doi:10.17843/rpmesp.2024.412.13373)
Supplement: Translation

# Translation: Adaptación y análisis psicométrico del cuestionario de ansiedad generalizada (GAD-7) al Quechua Collao en Perú

**Authors:** Julio Cjuno, Raúl Alexis Villegas-Mejía, Jovita Coronado-Fernández

**Affiliations:** 1 Universidad Peruana Unión, Escuela de Posgrado de Ingeniería y Arquitectura. Lima, Perú; 2 Universidad Católica Los Ángeles de Chimbote, Escuela de Enfermería. Chimbote, Perú

**Keywords:** Trastornos de ansiedad, ansiedad, GAD-7, pueblos originarios

## Abstract

**Objetivos.:**

Adaptar del inglés al contexto cultural y lingüístico del Quechua Collao y analizar las propiedades psicométricas del Cuestionario de Ansiedad Generalizada de 7 ítems (GAD-7) en Puno, Perú.

**Material y métodos.:**

El GAD-7 en su versión original fue traducido a la variedad del quechua Collao y se analizaron sus propiedades psicométricas, los participantes fueron bilingües (español y quechua) mayores de 18 años y de ambos sexos. Se evaluó el análisis factorial exploratorio (AFE) empleando el análisis paralelo, el análisis factorial confirmatorio (AFC) y los índices de bondad de ajuste; asimismo se analizó la fiabilidad mediante el alfa clásico y Omega de Mc Donald.

**Resultados.:**

Los jueces y participantes del grupo focal realizaron la adaptación cultural y lingüística del GAD-7 al Quechua Collao; el AFE reportó la presencia de un único factor (KMO=0,88, p=0,01); mientras que el AFC confirmó adecuados ajustes en el modelo unifactorial (CFI=0,994; TLI=0,991; SRMR=0,027; RMSEA=0,092), una buena confiabilidad (α=0,896; ω=0,894) y además fue invariante en grupos de edad, sexo, estado civil y grado de instrucción.

**Conclusiones.:**

El cuestionario presentó validez para un modelo unidimensional del GAD-7 adaptado al Quechua Collao, así como una óptima confiabilidad e invarianza por los grupos evaluados. Su uso podría beneficiar la investigación y atención de la ansiedad.

## INTRODUCCIÓN

La ansiedad es un trastorno mental frecuente con crecimiento constante que junto con la depresión afecta a más 264 millones de personas en el mundo ^1^. En la región de las Américas 2,1% de los habitantes padecen ansiedad; mientras que, en Perú se han reportado valores de 5,3% ^2^ y en las poblaciones de la sierra peruana del 16,2% ^3^. A pesar de ser un problema frecuente de salud mental, no es diagnosticado a tiempo y el tratamiento casi siempre es postergado, por una carencia de personal con dominio del idioma e instrumentos adecuados ^4^. Un ejemplo de ello son los quechuas, una población originaria en Perú, que a pesar de contar con una extensa población de 1,326,454 habitantes aproximadamente ^5^; no cuentan con instrumentos de tamizaje de la ansiedad contextualizados a su entorno cultural y lingüístico ^6^.

En ese sentido, resulta importante destacar que el General Anxiety Disorder (GAD-7) tienen una amplia preferencia en los diversos estudios psicométricos y que lo identifican como un instrumento prometedor para el tamizaje de la ansiedad ^7^, debido a que su construcción teórica se basó en los criterios de diagnóstico del DSM-IV para ansiedad general ^8^; diseñado en inglés por un equipo de investigadores de Estados Unidos ^9^. Actualmente, cuenta con adaptaciones que hacen posible su uso en el tamizaje de la ansiedad, por los diversos estudios de adaptación y validación en idiomas como el inglés ^10^, alemán ^11^, chino mandarín ^12^, español ^13^, portugués ^14^, español boliviano ^15^, español colombiano ^16^. En todos los estudios previos, el GAD-7 ha mostrado un comportamiento de un solo factor y adecuadas evidencias de validez y fiabilidad. 

En Perú, el modelo unifactorial del GAD-7 se puede utilizar en mujeres embarazadas que hablan español ^17^ y en jóvenes universitarios ^18^. La ausencia de mediciones de ansiedad dentro de las comunidades quechua hablantes en Perú deja un vacío en la comprensión de la salud mental en esta población, la cual constituye cerca del 13,6% del total de habitantes del país ^19^. Su omisión, limita la capacidad de identificar y abordar los problemas de salud mental dentro de estas comunidades; asimismo obstaculiza el desarrollo de intervenciones efectivas y culturalmente sensibles para la ansiedad. Dada la importancia de la salud mental en la calidad de vida y el bienestar de las personas ^20^, es fundamental que se lleven a cabo estudios que consideren la experiencia y expresión de la ansiedad en las comunidades quechua hablantes, a fin de garantizar una atención integral y equitativa para todos los grupos étnicos del país.

Frente a ello, en este estudio se tuvieron como objetivos, adaptar del inglés al contexto cultural y lingüístico del Quechua Collao el GAD-7 y analizar sus propiedades psicométricas como la validez de la estructura interna y su fiabilidad.

**MENSAJES CLAVE**

Motivación para realizar el estudio. El Perú concentra la mayor población quechua; sin embargo, la medición de la ansiedad no está validada al contexto cultural y lingüístico de la variedad Quechua Collao que se habla principalmente en Puno.

Principales hallazgos. La adaptación al Quechua Collao del Cuestionario de Ansiedad Generalizada de 7 ítems (GAD-7) reportó una adecuada validez interna, invarianza y una óptima fiabilidad.

Implicancias. El GAD-7 adaptado al Quechua Collao podría implementarse en la atención primaria de la salud para el tamizaje de los síntomas de ansiedad generalizada.

## MATERIAL Y MÉTODOS

### Diseño y contexto

El presente es un estudio psicométrico, desarrollado en la región de Puno en Perú, que alberga aproximadamente a 538 127 quechua hablantes de la variedad Quechua Collao (57,0% de pobladores de Puno), dedicados principalmente a actividades comerciales, turismo, agricultura y ganadería ^5^.

### Traducción

El GAD-7 en su versión original en inglés fue traducido directamente a la variedad del quechua Collao. Culminada la traducción, los dos traductores y un investigador quechua hablante se reunieron para discutir sobre las diferencias de las traducciones. Una vez resuelta las discrepancias y unificadas las traducciones, se procedió con la traducción inversa del quechua al inglés. Posteriormente, los traductores y el equipo de investigadores verificaron la traducción inversa junto con la primera traducción, afinando detalles y brindando su visto bueno a la versión final del GAD-7 Quechua Collao. 

### Adaptación cultural al Quechua Collao

Aplicando el método Delphi, se elaboró una ficha de adaptación cultural (Material suplementario: Anexo 1) con algunas preguntas abiertas para palabras no comunes en quechua como: “ansiedad” y “nervioso”. Adicionalmente, se buscó consultar el cambio de la categoría de respuesta “more than half the days” por “ashka p’unchaykuna” y sobre la relación entre el GAD-7 Quechua Collao con el DSM-V para el diagnóstico de ansiedad generalizada. Aquellas interrogantes fueron enviadas a través de un correo electrónico. Participaron tres psicólogos bilingües (v.g. hablan español y quechua), todos reportaron al menos un año de experiencia en el tratamiento psicoterapéutico de la depresión en pacientes quechua hablantes de la variedad Collao. La interacción entre cada experto y el equipo de investigación se dio en dos rondas de correos, además de una reunión vía Zoom con el equipo de investigación para poder absolver las sugerencias y llegar a un consenso. 

Posteriormente, se organizó un grupo focal en un entorno virtual con el propósito de verificar la claridad y comprensión de los ítems en la población objetivo, dicha reunión duró 60 minutos aproximadamente. Al inicio, previa obtención del consentimiento informado, se solicitó responder el GAD-7 Quechua Collao en una versión en línea compartida vía Google Forms. Luego, el moderador (psicólogo quechua hablante nativo con conocimiento de métodos cualitativos) invitó a los participantes a opinar sobre la claridad y comprensión de los ítems en un lenguaje habitual y sencillo para el quechua hablante. Participaron cinco personas quechua hablantes mayores de 18 años (tres mujeres y dos varones) que eran bilingües (hablan quechua y español), este proceso se muestra en el (Material suplementario: Anexo 2). 

### Participantes

El estudio contó con dos muestras; para el análisis factorial exploratorio (AFE) se aplicó la versión final del GAD-7 traducido al Quechua Collao, en una muestra no probabilística de 206 participantes, siguiendo las recomendaciones para análisis factoriales de 20 participantes por ítem, es decir, 7 ítems X 20 participantes resultando 140 participantes ^21^; mientras que para el análisis factorial confirmatorio (AFC) la muestra fue de 454 participantes, siendo este número superior al calculado por la aplicación: https://wnarifin.shinyapps.io/ss_sem_cfi_equal/, donde el tamaño de la muestra del AFC para el modelamiento de ecuaciones estructurales (SEM) espera una muestra de 414 participantes, empleando un poder estadístico del 95% y una tasa de pérdida del 10%. 

En ambas muestras se incluyeron participantes mayores de 18 años, de ambos sexos, que viven en entornos urbanos y rurales, ubicados en los departamentos de Puno y Cusco, que reportaron ser quechua hablantes bilingües (hablan quechua y español), con una formación académica suficiente como para leer en quechua (v.g. primaria incompleta como mínimo), se excluyeron a hablantes de otras variedades del quechua y aquellos que no brindaron su consentimiento informado. 

### Instrumento

El GAD-7 en su versión original en inglés ^22^, consta de 7 ítems que se corresponden con los síntomas de ansiedad generalizada del DSM-IV ^8^. Sus opciones de respuesta evocan la frecuencia de aparición de tales síntomas en las últimas dos semanas, considerando la siguiente escala tipo Likert: 0 = not at all (para nada), 1 = several days (algunos días), 2 = more than half the days (varios días), 3 = nearly every day (casi todos los días). Esto define una puntuación cruda entre 0 y 21. La versión en inglés del GAD-7 mostró una sensibilidad y especificidad de 86 y 82% respectivamente, de forma similar reportó una validez basada en la relación con otras variables como Ansiedad de Beck (r = 0,72) y la subescala de ansiedad del Symptom Checklist-90 (r = 0,74) indicando una validez externa; así mismo, mostró una buena fiabilidad de α = 0,92.

### Procedimientos

Para la recolección de datos se capacitó en el uso y aplicación del instrumento a dos encuestadores, quienes fueron estudiantes de psicología del quinto año de estudios. Los encuestadores identificaron grupos de WhatsApp de padres de familia de instituciones educativas, iglesias cristianas y grupos de asociaciones de comunidades campesinas a quienes presentaron la encuesta en formato Google Forms, dicha encuesta inicialmente presentó el consentimiento informado; solo quienes aceptaron participar en el estudio pudieron responder a los ítems. La recolección de datos inició en enero y terminó en febrero del 2023.

### Análisis estadístico

Se realizaron los análisis descriptivos de los ítems (media, desviación estándar, asimetría y curtosis). Posteriormente se realizó el Análisis Factorial Exploratorio (AFE) donde se verificó el número de factores a través del análisis de paralelo, adicionalmente se estimó la prueba de Kaiser-Meyer-Olkin (KMO) a través de la fuerza de las correlaciones parciales entre las variables considerando un KMO > 0,80 como la adecuada plausibilidad; adicionalmente el test esfericidad de Bartlett (p < 0,05) deberá afirmar que la matriz de correlación no es una matriz de identidad ^23^. 

Asimismo, se realizó un Análisis Factorial Confirmatorio (AFC) del modelo unidimensional utilizando un estimador WLSMV (Weighted Least Square Mean and Variance Adjusted), tal y como lo hicimos previamente ^24^. Reportamos los betas estandarizados del modelo y las medidas estándar de bondad de ajuste: el X2 para modelo versus línea de base, considerando aceptable valores < 3; el índice de ajuste comparativo (CFI), que es adecuado cuando es > 0,90; el índice de Tucker-Lewis Index (TLI), que es aceptable cuando es > 0,90. Asimismo, el residuo estandarizado cuadrático medio (SRMR) y la raíz del error cuadrático medio de aproximación (RMSEA), considerándolos adecuados con valores ≤ 0,08 ^25^. Se empleó el Modelamiento de Ecuaciones Estructurales SEM para poder presentar de forma gráfica las cargas factoriales ^26^ y finalmente se estimó la consistencia interna empleando el Alfa clásico y el Omega de Mc Donald’s ^27^.

La invarianza de medida se analizó por medio del método de CFA Multigrupos considerando grupos definidos por sexo y estado civil. La diferencia en el CFI (ΔCFI) o RMSEA (ΔRMSEA) el criterio para determinar los modelos con más restricciones versus los que tenían menos restricciones, asumiendo inicialmente la invarianza configurar como modelo base, escalando a la invarianza métrica, fuerte y estricta. Para poder determinar un modelo adecuadamente restringido y apropiado en cada etapa de invarianza los ΔCFI o ΔRMSEA debieron presentar valores < 0,01 ^28^.

Adicionalmente se realizaron modelos MIMIC (Múltiples Indicadores y Múltiples Causas) para determinar la invarianza por edad y grado de instrucción (v.g. debido a que fueron variables numéricas o cuando no fue viable emplear el CFA Multigrupos). En ello, se evaluaron las invarianzas de los interceptos de los indicadores y las diferencias de media de las dimensiones latentes, todo ello a través de grupos según las variables ya antes mencionadas. Cada covariable fue evaluada por separado, comparando cada uno de ellos dos tipos de modelos; la primera es una versión saturada donde la covariable explica todos los ítems observados, pero no las dimensiones latentes y el segundo, una versión del modelo de invariante intercepto donde la covariable explica todas las dimensiones latentes, pero no los ítems. Para determinar un modelo más restringido tuvimos que identificar un ΔCFI o un ΔRMSEA <0,01 ^28^. Todo ello empleando el software estadístico R Studio versión 3,3,0+ y sus paquetes psych, GPArotation, polycor, lavaan, semTools y semPlot.

### Consideraciones éticas

Este estudio fue evaluado y aprobado por el Comité de Ética de la Universidad Peruana Unión con informe Número 2022-CE-FCS - UPeU-059. Asimismo, se respetaron todos los principios éticos de investigación en seres humanos de la declaración de Helsinki ^29^.

## RESULTADOS

### Fase de adaptación cultural

Los expertos que participaron en la adaptación cultural por el método Delphi; brindaron valiosas sugerencias que contribuyeron a mejorar la adaptación de la palabra “ansiedad” donde inicialmente estuvo adaptada como *“*phutisk’alla”, se sugirió agregar “ansiedad nisqhawan”, si bien es una combinación entre español y quechua esto ayuda a entenderlo mejor, quedando como “phutisk’alla/ansiedad nisqhawan” en el ítem 1. Mientras que, para la expresión “nervioso” los expertos recomendaron implementar la pregunta cómo “Ancha mancharisqha”. Por otro lado, los participantes del grupo focal brindaron una opinión favorable respecto a la claridad y comprensión del GAD-7 Quechua Collao, aseverando que es claro y acorde a su contexto cultural sin más cambios adicionales.

Por otro lado, las opciones de respuesta (tipo Likert) del GAD-7 Quechua Collao también fueron cambiadas por los jueces expertos y los participantes del grupo focal; esto debido a que la categoría “más de la mitad de los días” presentó problemas de claridad en su traducción. La recomendación final del equipo fue utilizar las expresiones “Mana hayk’aqpas”, “Wakin p’unchawkunalla”, “Ashka p’unchawkuna”, “Yaqa llapa p’un chawkuna”, representando el equivalente en español de “nunca”, “algunos días”, “varios días”, “casi todos los días”. Los expertos que participaron en la adaptación cultural; después de las mejoras implementadas en los ítems y categorías de respuesta del GAD-7 Quechua Collao, en una última interacción con el equipo de investigación, calificaron con la puntuación máxima cada ítem (3/3 en un rango de 0 a 3), en cada indicador de validez de contenido como: relevancia, representatividad, claridad y equivalencia cultural. 

### Características de los participantes

La muestra del AFE estuvo conformada por 206 participantes con una edad promedio de 31 años, de los cuales el 57,3% (118) fueron de sexo femenino, el 6,1% (132) fueron soltero/viudo/divorciado y el 59,7% (123) reportaron tener estudios universitarios; mientras que la muestra del AFC estuvo conformada por una muestra de 454 participantes con una edad promedio de 32 años, donde el 57,7% (262) fueron de sexo femenino, 57,0% (259) reportaron ser soltero/viudo/divorciado y el 40,7% (185) indicaron tener estudios universitarios (Material suplementario: Anexo 3).

### Análisis factorial exploratorio

El gráfico de sedimentación o del análisis paralelo, reportó la presencia de un único factor latente (Figura 1). Mientras que la prueba de Kaiser-Meyer-Olkin y test de esfericidad de Bartlett (KMO = 0,88, p = 0,01) confirmó la presencia de aquel factor a partir de las variables incluidas (Material suplementario: Anexo 3) con pesos factoriales que oscilan entre (0,64 - 0,85) obtenido por el método de Máxima verosimilitud y una rotación varimax; indicando que teóricamente los ítems forman un factor (Tabla 1).

**Figura 1:** Análisis paralelo del GAD-7 adaptado al Quechua Collao

**Tabla 1:** Análisis descriptivo de los ítems del GAD-7 adaptado al Quechua Collao.

Ítems	Media	Desviación estándar	Asimetría	Curtosis	Carga factorial (1 factor)
Pregunta 1	0,78	0,81	0,87	0,30	0,65
Pregunta 2	0,81	0,83	0,89	0,28	0,85
Pregunta 3	0,93	0,80	0,76	0,40	0,75
Pregunta 4	0,82	0,83	0,88	0,29	0,80
Pregunta 5	0,69	0,83	1,16	0,85	0,66
Pregunta 6	0,98	0,87	0,62	-0,28	0,64
Pregunta 7	0,71	0,82	1,07	0,61	0,82

### Análisis factorial confirmatorio

El modelo de un solo factor reportó adecuados valores de bondad de ajuste para el GAD-7 Quechua Collao, (CFI = 0,994; TLI = 0,991; SRMR = 0,027; RMSEA = 0,092) (Tabla 2). En cuanto a las cargas factoriales, se obtuvo un mínimo de λ = 0,72 y un máximo de λ = 0,86 en los ítems del GAD-7 Quechua Collao (Figura 2). 

**Tabla 2:** Análisis Factorial Confirmatorio con estimador WLSMV para el GAD-7 adaptado al Quechua Collao

Modelo	Índice de bondad de ajuste	
1 dimensión	X^2^ (14)	0,181
	Índice de ajuste comparativo (CFI)	0,994
	Índice de Tucker-Lewis (TLI)	0,991
	Residuo cuadrático medio estandarizado (SRMR)	0,027
	Error cuadrático medio de aproximación (RMSEA)	0,092
	Alpha	0,896
	Omega	0,894

X^2^ (df>) Versus la línea de base

**Figura 2:** Gráfico SEM del modelo unifactorial del GAD-7 adaptado al Quechua Collao con betas y errores.

### Confiabilidad

Adicionalmente, el GAD-7 Quechua Collao reportó una buena confiabilidad, con valores de Alfa de Cronbach de 0,896 y Omega de 0,894 (Tabla 2).

### Invarianza de medida

El análisis CFA multigrupo para GAD-7 Quechua Collao, ha determinado invarianza de medida según sexo y estado civil, presentando adecuados índices de bondad de ajuste (CFI > 0,98, RMSEA < 0,08); así también, considerando valores < 0,01 para al menos al ΔCFI o ΔRMSEA; luego de verificar la invarianza configural, se analizó la invarianza métrica (ΔCFI = 0,002), invarianza fuerte (ΔCFI = 0,003) e invarianza estricta (ΔCFI < 0,004) (Tabla 3). Por otro lado, por medio de la Invarianza de Múltiples Indicadores y Múltiples Causas (MIMIC) se confirmó la invarianza por edad y grado de instrucción puesto que presentaron adecuados índices de bondad de ajuste (CFI > 0,99, TLI > 0,99, RMSEA < 0,07, SRMR < 0,02) y los ΔCFI, ΔTLI, ΔMRSEA todos fueron < 0,01 en la invarianza saturado y de intercepto (Tabla 4).

**Tabla 3:** Invarianza CFA multigrupo según sexo y estado civil para el GAD-7 adaptado al Quechua Collao

Variable	Invarianza	X^2^	gl	Valor p	CFI	Δ CFI	RMSEA	Δ RMSEA
Sexo	1. Configural	31,742	28	-	0,992	-	0,079	-
	2. Métrica	37,643	34	0,665	0,994	0,002	0,062	0,017
	3. Fuerte	52,986	47	0,028	0,991	0,003	0,064	0,002
	4. Estricta	57,715	54	0,831	0,992	0,002	0,055	0,009
Estado civil	1. Configural	30,754	28	-	0,992	-	0,078	-
	2. Métrica	49,397	34	0,035	0,990	0,002	0,080	0,002
	3. Fuerte	47,144	47	1,000	0,993	0,003	0,057	0,023
	4. Estricta	71,242	54	0,008	0,989	0,004	0,067	0,010

X²(gl) = Estadístico de chi-cuadrado (χ²) con grados de libertad (gl), CFI = Índice de Ajuste Comparativo, TLI = Índice Tucker-Lewis, SRMR = Residual Estandarizado de la Raíz Cuadrada Media, RMSEA = Error Cuadrático Medio de Aproximación, Δ = diferencia.

**Tabla 4:** Invarianza de Múltiples Indicadores y Múltiples Causas (MIMIC) por edad y grado de instrucción

Covariantes	Modelo	CFI	TLI	RMSEA	SRMR	Δ CFI	Δ TLI	Δ MRSEA
Edad	MIMIC saturado	0,994	0,991	0,068	0,027	-	-	-
	Invarianza MIMIC intercepto	0,992	0,992	0,064	0,027	-0,002	0,001	-0,004
Grado de Instrucción	MIMIC saturado	0,991	0,990	0,070	0,027	-	-	-
	Invarianza MIMIC intercepto	0,994	0,990	0,069	0,027	0,003	0,000	-0,001

CFI = Índice de Ajuste Comparativo, TLI = Índice Tucker-Lewis, SRMR = Residual Estandarizado de la Raíz Cuadrada Media, RMSEA = Error Cuadrático Medio de Aproximación, Δ = diferencia

## DISCUSION

El GAD-7 adaptado al Quechua Collao mostró adecuados ajustes para un modelo unifactorial, asimismo fue invariante por grupos de sexo, estado civil, edad y grado de instrucción; además presenta una confiabilidad óptima para su uso en quechua hablantes de la variedad Collao. El uso del GAD-7 Quechua Collao podría ser útil para evaluar los síntomas de ansiedad en población quechua hablante extendida por los departamentos de Puno, Cusco, Arequipa, Moquegua, Tacna y Puerto Maldonado.

Desde el análisis gráfico de paralelos y cargas factoriales en el AFE y los índices de bondad de ajuste en el AFC apoyaron un modelo unifactorial; aunque el RMSEA presentó valores un tanto elevados pero no al punto de llegar a un desajuste ^30^; el RMSEA usualmente tolera cierto desajuste por el tamaño de muestra ^31^. Sin embargo, aquel comportamiento no solo se ha presentado en nuestro estudio, los estudios realizados en Alemania ^11^, España ^13^, Brasil ^14^, Colombia ^16^ y Bolivia ^15^ también presentaron valores elevados. A pesar de la tendencia elevada en el RMSEA en los diversos estudios, en nuestro caso podría atribuirse al tamaño de la muestra ^30^ y las diferencias de proporciones de submuestras principalmente por grado de instrucción. Otra razón podría ser la inclusión de personas sin estudios, muy a pesar de que sabían leer, es posible que algunos ítems no sean fáciles de comprender para ellos; estudios posteriores podrían buscar adaptar el instrumento en un formato audible para personas nativos quechua hablantes sin formación escolarizada. A pesar de ello, el GAD-7 Quechua Collao es un instrumento de tamizaje válido con una estructura unifactorial, debido a que los otros índices de bondad de ajuste cumplieron estrictamente los parámetros esperados.

Nuestros hallazgos reportan una confiabilidad óptima de los puntajes derivados del GAD-7 Quechua Collao en población peruana. Aquello es consistente con otros hallazgos, en estudios validación del GAD-7 previos como el de Alemania (α = 0,85) ^11^, China (α = 0,84) ^12^, España (α = 0,94) ^13^, Brasil (α = 0,92) ^14^, Colombia (α = 0,92) ^16^ quienes identificaron valores de fiabilidad similares a los que encontramos. A pesar de ser estudios en poblaciones cultural y lingüísticamente diferentes las medidas de la consistencia interna por el alfa clásico presentan una consistencia similar. Aquello, otorga una característica de comparabilidad de la variable ansiedad intercultural al haber logrado un instrumento óptimo en su consistencia interna para poblaciones quechua hablantes de la variedad Collao. En cuanto a la elección del Alfa Clásico para estimar la fiabilidad posiblemente se deba a la búsqueda de la comparabilidad de los datos para fines de meta-analizarlas ^32^. En nuestro estudio adicionalmente estimamos el Omega McDonald con la intención de evaluar la consistencia interna desde las cargas factoriales, por tratarse de una opción más adecuada de estimación de la fiabilidad ^27^, sin ser afectados por el número de ítems o categorías de respuesta, como sucede en el alfa clásico ^33^. Contar con óptimos valores de confiabilidad indica que esta versión del GAD-7 es consistente y confiable para medir la ansiedad en quechua hablantes de la variedad Collao; aquello, debido a que los ítems del constructo están correlacionados entre sí de manera consistente en la medición del constructo subyacente, pudiendo así ofrecer mediciones confiables y consistentes.

Así también el GAD-7 Quechua Collao, ha presentado invarianza configural, métrica, escalar y estricta para grupos de sexo y estado civil; asimismo, reportó invarianza saturada y de intercepto por edad y grado de instrucción. Estudios previos también mostraron resultados similares, como el realizado en Italia donde el GAD-7 fue invariante por edad y género ^34^; mientras que en China presentó evidencias de invarianza por edad, sexo, nivel de educación y residencia ^35^; asimismo, otro estudio realizado en Reino Unido, Irlanda, España e Italia encontró invarianza saturada y de intercepto por grupos de país, edad y sexo ^36^. Aquellas similitudes entre los estudios previos y los nuestros, sugieren que en quechua hablantes de la variedad Collao, el GAD-7 Quechua Collao permite realizar comparaciones de la medida de ansiedad sin variación en la medición entre varones y mujeres, casado/conviviente y solteros/viudos/divorciados, grupo de edad y en aquellas personas sin estudios, con educación primaria, secundaria, superior técnico y universitaria.

El presente estudio reporta las siguientes limitaciones como la técnica de recolección de datos por autorreporte, que pudo influir en que los participantes sean poco honestos al momento de responder los ítems; sin embargo, previamente se sensibilizó a los participantes sobre la importancia de ser honestos al momento de responder el instrumento. Por otro lado, es posible que la encuestadora no haya respetado el protocolo de aplicación por autorreporte, pudiendo en algunos casos adicionar explicaciones a consultas de los participantes ello pudo influir en los valores un tanto elevados del RMSEA; para ello buscamos controlar el efecto del evaluador con una capacitación del proceso de presentación y recolección de datos, asimismo se realizó un seguimiento constante sobre su función para que se respete la técnica de recolección por autorreporte. También resulta importante declarar, que los modelos MIMIC que empleamos para la evaluación de invarianza por edad y grado de instrucción, se limitan a evaluar los modelos de intercepto invariante y las medias factoriales y, asume que el resto de los parámetros estructurales y de medida como las cargas factoriales, error de varianza o covarianza, factor de varianza o covarianza son los mismos a través de todos los niveles de estas variables.

En conclusión, los hallazgos muestran la validez para un modelo unidimensional del GAD-7 Quechua Collao, con óptima confiabilidad e invarianza por los grupos evaluados como edad, sexo, estado civil y grado de instrucción. Su uso podría beneficiar la investigación y atención de la ansiedad en población quechua hablante procedente de la región Puno en Perú.
